# Incremental decreases in quality-adjusted life years (QALY) associated with higher levels of depressive symptoms for U.S. Adults aged 65 years and older

**DOI:** 10.1186/s12955-016-0582-8

**Published:** 2017-01-11

**Authors:** Haomiao Jia, Erica I. Lubetkin

**Affiliations:** 1 Department of Biostatistics, Mailman School of Public Health and School of Nursing, Columbia University, 617 West 168th Street, New York, NY 10032 USA; 2 Department of Community Health and Social Medicine, CUNY School of Medicine, New York, NY USA

**Keywords:** Quality-adjusted life year (QALY), Health-related quality of life (HRQOL), Burden of disease, Depression, Major depressive disorder (MDD)

## Abstract

**Background:**

Quality-adjusted life years (QALY) is a single value index that quantifies the overall burden of disease. It reflects all aspects of heath, including nonfatal illness and mortality outcomes by weighting life-years lived with health-related quality of life (HRQOL) scores. This study examine the burden of disease due to increasing levels of depressive symptoms by examining the association between the 9-item Patient Health Questionnaire (PHQ-9) scores and QALY for U.S. adults aged 65 years and older.

**Methods:**

We ascertained respondents’ HRQOL scores and mortality status from the 2005–2006, 2007–2008, and 2009–2010 cohorts of the National Health and Nutrition Examination Survey (NHANES) with mortality follow-up data through December 31, 2011. This analysis included respondents aged 65 years and older (*n* = 3,680). We estimated the mean QALY throughout the remaining lifetime according to participants’ depression severity categories: none or minimal (PHQ-9 score 0–4), mild (5–9), moderate (10–14), and moderately severs and severe (15 or higher). We estimated QALY loss due to major depressive disorder (PHQ-9 score 10 or higher) and to mild depression (5–9).

**Results:**

The QALY for persons with none/minimal, mild, moderate, and moderately severe/severe depression were 14.0, 7.8, 4.7, and 3.3 years, respectively. Compared to persons without major depressive disorder, persons with major depressive disorder had 8.3 fewer QALY (12.7 vs. 4.4), or a 65% loss. Compared to persons who reported “none” or minimal depressive symptoms, persons who reported mild depressive symptoms had 6.2 fewer QALY (14.0 vs. 7.8), or a 44% loss. The same patterns were noted in demographic and socioeconomic subgroups and according to number of comorbidities.

**Conclusions:**

This study not only confirmed the significant burden of disease for major depressive disorder among the U.S. elderly, but also showed an incremental decrease in QALY with an increasing severity of depressive symptoms as well as significant QALY loss due to mild depression. Specifically, individuals with higher (or more impaired) PHQ-9 scores had significantly fewer QALYs and our findings of fewer years of QALY for persons with major depressive disorder and mild depression were not only statistically significant but also clinically important.

## Background

Depression is a prevalent condition and is an important public health problem in the United States [1–3]. In large nationally representative surveys the prevalence of depression was estimated to be 6.7% in the past 12 months and 16.6% over a lifetime [4]. Depression often is associated with other comorbid conditions and may worsen their health outcomes [5]. Depression can also be life threatening and has been associated with excess mortality and substantially lower life expectancy [6, 7]. In a recent study of the U.S. adult population, individuals with depression lost a remarkable 16.4 years of life relative to those without depression [7].

In the United States and throughout the rest of the world, depression has been considered to be an important contributor to the burden of disease. The Global Burden of Disease Study estimated disability-adjusted life years (DALYs) worldwide and found that depression was the leading health condition worldwide in terms of DALYs, contributing 917 DALY per 100,000 persons annually [8]. Jia and colleagues estimated quality-adjusted life expectancy (QALE) for U.S. adults and found that depression led to a 28.9-year QALE loss at age 18, a number that greatly exceeded the QALE loss for many other chronic conditions and risky lifestyle behaviors such as smoking and physical inactivity [7].

In the elderly, reports of the prevalence of depression among the non-institutional population range from approximately 8 to 16% [9]. At age 65, those with major depressive disorder lost 13.8 years of QALE [7]. Understanding the depression associated burden of disease would be particularly important in the elderly, given that the number of persons 65 and older in the United States is projected to nearly double between 2012 and 2050 and depression is more common among persons with chronic conditions and functional limitations [10, 11]. Depression may be more difficult to detect in the elderly due to a different clinical presentation and a greater likelihood to present in the context of these comorbid medical conditions [3]. Additionally, population-based studies have indicated that mean psychological distress symptoms have not decreased over time, despite increasing use of health services [12]. With regard to treatment, older depressed patients may be undertreated compared with younger adults [13]. Yet, over 80% of elderly depressed outpatients without significant comorbid medical illness or dementia who are optimally treated may recover and remain well during follow-up [9, 14].

Like many other chronic conditions, the severity of depression can range from mild to moderate to severe [15, 16]. Clinicians and investigators have constructed different definitions of depression and administered a variety of different instruments for surveillance and diagnosis [15, 17]. For example, the 9-item Patient Health Questionnaire (PHQ-9) is a valid diagnostic and severity measure for depressive disorder in large clinical studies and for tracking depression prevalence in representative surveys of the U.S. general population [15]. The PHQ-9 consists of the nine criteria from which the diagnosis of depressive disorders is based [16]. Major depressive disorder (MDD) or clinical depression is defined as a score of 10 or higher [15]. The PHQ-9 cut-off of 10 for MDD includes moderate, moderately severe, and severe depression. By contrast, mild depression is considered to be a PHQ-9 score of between 5 and 9. The majority of persons characterized with depressive symptoms have mild depression and, for this group, the recommendation is watchful waiting and reassessment for antidepressant treatment or psychotherapy after three months [15]. For The Global Burden of Disease Study, the investigators specified that mental disorders had to meet the threshold for a case according to criteria described in the Diagnostic and Statistical Manual of Mental Disorders (DSM) or the International Classification of Diseases (ICD) [8, 18]. Although the Global Burden of Disease study modeled different severity levels for DSM or ICD diagnosed depression, this study did not examine the incremental impact of different severity levels or estimate the burden of disease for persons with mild depression.

The main goal of the current study is to estimate the burden of disease attributable to different levels of depressive symptoms for U.S. adults aged 65 years and older. Specifically, we estimated mean quality-adjusted life years (QALY) throughout the remaining lifetime according to respondents’ PHQ-9 scores, and, by doing so, we estimated the decreases in QALY (i.e., QALY loss) for those with major depressive disorder (MDD) as compared to those without MDD, and for those with mild depressive symptoms as compared to those with none or minimal depressive symptoms. We also estimated the QALY losses due to MDD and to mild depression according to demographic and socioeconomic subgroups and according to number of comorbidities.

## Methods

Quality-adjusted life years (QALY) is a single value index that quantifies the burden of disease. It reflects all aspects of heath, including nonfatal illness and mortality outcomes, by weighting life-years lived with preference-based health-related quality of life (HRQOL) scores [19]. Preference-based HRQOL, also called health utility value, is a summary score that assesses the values of one health state vs. another state. The health utility value is anchored at 0 for death and 1 for perfect health, so one year lived in a reduced health state of utility value of 0.5 is equal to 0.5 QALYs, the same as lived one half year in perfect health [19]. In this analysis, we calculated mean QALY throughout the remaining lifetime for participants according to their PHQ-9 scores.

### Data

We ascertained respondents’ HRQOL scores and mortality status from the 2005–06, 2007–08, and 2009–2010 cohorts of the National Health and Nutrition Examination Survey (NHANES) Linked Mortality File [20, 21]. The NHANES is an ongoing survey of random samples from the non-institutionalized civilian population of the U.S. [20]. With the use of the design weight and adjustment for noncoverage and nonresponse, the distribution of respondents was representative of the U.S. general population [20]. The NHANES Linked Mortality File was created by the National Center for Health Statistics (NCHS) by linking the NHANES respondents to the National Death Index (NDI) [21]. The respondents in this analysis had mortality follow-up through December 31, 2011. We included only respondents aged 65 years and older at the baseline, yielding a total sample size of 3,680.

### Measures

The NHANES has included the PHQ-9 since the 2005–2006 cohort [20]. The PHQ-9 asks questions about the frequency of symptoms of depression over the past two weeks. In the PHQ-9 response categories “not at all,” “several days,” “more than half the days,” and “nearly every day” are given a score ranging from 0 to 3. A total score is calculated ranging from 0 to 27. The PHQ-9 can be used to classify depressive symptoms into five severity categories: none or minimal (0–4), mild (5–9), moderate (10–14), moderately severe (15–19), and severe (20–27) [15]. Major depressive disorder (MDD) is defined as having a PHQ-9 score of 10 or higher and mild depression is defined as having a PHQ-9 score of 5–9 [15].

The NHANES asks respondents to rank their general health from 1 (excellent) to 5 (poor) and to report numbers of their physically unhealthy days, mentally unhealthy days, and days with activity limitation during the past 30 days [22]. This study employs a previously constructed mapping algorithm based on respondents’ age and answers to these four questions to obtain values of a frequently used preference-based HRQOL measurement, the EQ-5D index, to calculate QALY [23]. This algorithm provides valid estimates of EQ-5D scores for respondents [23, 24], and the bias of estimated scores has been estimated to be less than 1% of that using the actual EQ-5D questions [24].

The NHANES includes information on respondent sociodemographic characteristics and certain diseases at the baseline [20]. These variables were included in the analyses of the depression outcome to assess potential associations with these variables. The analysis examined age, gender, race/ethnicity, education achievement, income, marital status, and number of comorbidities. The NHANES calculated respondents’ family income to the Federal Poverty Level (FPL) ratio. We used 138% FPL, the Medicaid income eligibility limit, as the cut-off point for income.

### Statistical analysis

Calculation of mean QALY throughout the remaining lifetime is difficult because most of the participants were alive at the end of follow-up [25]. It requires extrapolating quality-adjusted survival time beyond the end of follow-up. This study proposed and applied a hybrid method that calculated QALY from two parts: QALY during the follow-up period (to December 31, 2011) and QALY beyond the follow-up period (after December 31, 2011). Details of this method were described previously [25]. To summarize: QALY during the follow-up period was estimated based on the Kaplan-Meier method [25, 26]. Let *L* be the time of the end of follow-up and 0 < *t*_1_ ≤ *t*_2_ ≤ … ≤ *t*_*l*_ < *L* be times when deaths occurred. Suppose *Ŝ*_*KM*_(*t*) is the Kaplan-Meier estimated survival function. We calculate mean QALY at ${t}_i\ \left(i=1, \dots,\ l\right),\;\widehat{Q}\left({t}_i\right)$, for those who died at *t*_*i*_; and at time L, $\widehat{Q}(L)$, for who were alive at the end of follow-up. QALYs for time period (0, L] was estimated as:

$$ \left[{\displaystyle \sum_{j=1}^l}\widehat{Q}\left({t}_j\right)\left({\widehat{S}}_{KM}\left({t}_{j-1}\right)-{\widehat{S}}_{KM}\left({t}_j\right)\right)\right]+\widehat{Q}(L){\widehat{S}}_{KM}\left({t}_l\right), $$

where *t*_0_ = 0 and *S*(*t*_0_) = *S*(0) = 1.

The QALY beyond the follow-up period was estimated by extrapolating survival time beyond the end of follow-up. Because the model usually fits data well during the early follow-up but does not fit data well near the end of the follow-up, the model may not extrapolate the survival function well in the tail [27]. Instead, we used the parametric method to estimate total expected life-years and the Kaplan-Meier method to estimate life-years from time 0 to L. We used the Weibull model, *S*_*p*_(*t*) = exp[−(*t*/*λ*)^*β*^] and the QALYs in the tail was estimated as:

$$ \widehat{q}(L)\left\{\left[\widehat{\lambda}\Gamma \left(1+\frac{1}{\widehat{\beta}}\right)\right]-\left\{\left[{\displaystyle \sum_{i=0}^{t_{k-1}}}{\widehat{S}}_{km}\left({t}_i\right)\left({t}_{i+1}-{t}_i\right)\right]+{\widehat{S}}_{km}\left({t}_k\right)\left(L-{t}_k\right)\right\}\right\} $$

where Γ(*t*) = ∫_0_^∞^ *x* ^*t* − 1^ *e* ^− *x*^ *dx* is the Gamma function.

The QALY loss due to MDD was defined as the difference in QALY for participants without MDD and for participants with MDD [7, 25]. Similarly, the QALY loss due to mild depression was defined as the difference in QALY for participants who reported none or minimal depressive symptoms (PHQ-9 scores of 0–4) and for participants with mild depression. A propensity score method was used to account for the systematic difference in participants’ characteristics, such as age and sex, between those with different levels of depressive symptoms [28].

## Results

The average age of the population was 73.3 years (SD = 5.7 years) at the baseline (Table 1). Women comprised 55% of the population and non-Hispanic whites comprised 84% of the population. Only 8% were non-Hispanic blacks and 6% were Hispanics. In this population, the mean EQ-5D score was 0.827 (Table 2). About 12.6% of participants died during the follow-up, yielding a mortality rate of 3.51 deaths per 100 person-years. The mean QALY throughout the remaining lifetime was 12.3 years (10.3 years for men and 14.4 years for women).

**Table 1:** Baseline Characteristic, 2005–2010 NHANES

	Number	Percent^a^	S.E.
Total	3,680	100.0%	-
Age: mean (SD)	3,680	73.3 (5.7)	
65–74	1,979	57.6%	1.3%
75+	1,701	42.4%	1.3%
Sex			
Men	1,866	44.6%	0.8%
Women	1,814	55.4%	0.8%
Race			
Non-Hispanic whites	2,338	83.5%	1.5%
Non-Hispanic blacks	615	8.0%	0.9%
Hispanics	635	5.8%	0.9%
Other	92	2.6%	0.5%
Income			
<138% FPL^b^	500	8.9%	0.6%
≥138% FPL	2,877	91.1%	0.6%
Education			
≤High school	2,269	54.7%	2.0%
>High school	1,404	45.3%	2.0%
Married or with partner			
Yes	2,071	60.7%	1.3%
No^c^	1,609	39.3%	1.3%
Co-morbidities			
0 or 1	1,139	35.7%	1.6%
2 or more	2,341	64.3%	1.6%
PHQ-9 Score			
0–4	2,863	82.1%	0.8%
5–9	494	13.8%	0.6%
10–14	122	3.2%	0.3%
15–19	43	0.8%	0.1%
20–27	8	0.2%	0.1%

^a^ Weighted percent, accounted for sampling design, noncoverage, and nonresponse

^b^ Federal Poverty Level, where 138% FPL is the Medicaid income eligibility limit

^c^ Divorced, separated, never married, widowed

**Table 2:** EQ-5D index, Mortality Rate, and Quality-adjusted Life Years (QALY) throughout remainder of lifetime by Depressive Symptom Severity Categories, U.S. Adults Aged 65 Years and Older

PHQ-9 score	Depression severity categories	Number	EQ-5D^a^	S.E.	Mortality rate^b^	S.E.	QALY^c^	S.E.
0–27	Total	3,680	0.827	0.005	3.51	0.18	12.3	1.1
0–4	None-minimal	2,863	0.875	0.004	2.99	0.18	14.0	1.4
5–9	Mild	494	0.680	0.018	4.69	0.60	7.8	1.0
10–14	Moderate	122	0.482	0.038	4.97	1.11	4.7	0.7
15–27	Moderately severe/ severe	51	0.353	0.063	8.15	2.81	3.3	1.3

^a^ EQ-5D index, adjusted for age and sex in subgroups

^b^ Mortality rate per 100 person-years, adjusted for age and sex in subgroups

^c^ Quality-adjusted life years, adjusted for age and sex in subgroups

Among U.S. adults aged 65 years and older, 82.1% of participants had none or minimal depressive symptoms, 13.8% had mild depression, and 4.1% had MDD (ranging from moderate to moderately severe to severe). Because only 8 participants had a PHQ-9 score in the range of severe depressive disorder (20 or higher), we combined those with a PHQ-9 score of 20 or higher with those having a PHQ-9 ranging from 15–19. Mean EQ-5D scores decreased as the severity of depressive symptoms increased and mortality rates increased with increasing severity of depressive symptoms (Table 2). The mean QALY also decreased in a predictable manner according to the severity of depression. In particular, the QALY for those with none/minimal, mild, moderate, and moderately severe to severe depression were 14.0, 7.8, 4.7, and 3.3 years, respectively.

When the severity of depression was categorized according to the MDD status, the QALY were 4.4 years for persons with MDD and 12.7 years for persons without MDD (Table 3). This represents a decrease in QALY of 8.3 years, or a loss of 65% QALY, for those with MDD as compared to those without MDD. In subcategories of MDD, QALY also decreased with a higher level of depressive severity. Specifically, moderate depression contributed a loss of 8.0 QALYs (63%), and moderately severe to severe depression contributed a loss of 9.4 QALYs (74%).

**Table 3:** Decrease in Quality-adjusted Life Years (QALY) throughout remainder of lifetime due to Major Depressive Disorder (MDD) and to Mild Depression, U.S. Adults Aged 65 Years and Older

PHQ-9 score	Depression severity categories	Number	QALY^a^	S.E.	Loss^b^	S.E.	% Loss
0–9	No MDD	3,357	12.7	1.1	Ref	-	-
10–27	MDD^c^	173	4.4	0.9	8.3	1.2	65%
	*Subcategories of MDD*						
10–14	Moderate	122	4.7	0.7	8.0	1.1	63%
15–27	Moderately severe/severe	51	3.3	1.3	9.4	1.6	74%
0–4	None-minimal	2,863	14.0	1.4	Ref	-	-
5–9	Mild depression	494	7.8	1.0	6.2	1.3	44%
5–27	Mild depression or MDD	667	6.5	0.8	7.5	1.3	54%

^a^ Quality-adjusted life years (QALY) throughout remainder of lifetime, adjusted for age and sex in subgroups

^b^ Decrease in QALY for higher levels depressive symptoms vs. lower level depressive symptoms

^c^ Major depressive disorder (MDD)

Among persons without MDD, persons with mild depression had significantly lower QALY than those with none or minimal depressive symptoms (7.8 vs. 14.0 QALYs), or a loss of 6.2 QALYs (44%) for those with MDS as compared to those with none or minimal depressive symptoms. For those with any depression (having mild depression or MDD), QALY was 6.5 years. Therefore, any depression contributed a loss of 7.6 QALYs (55%) as compared to those with none or minimal depressive symptoms.

**Fig. 1:**
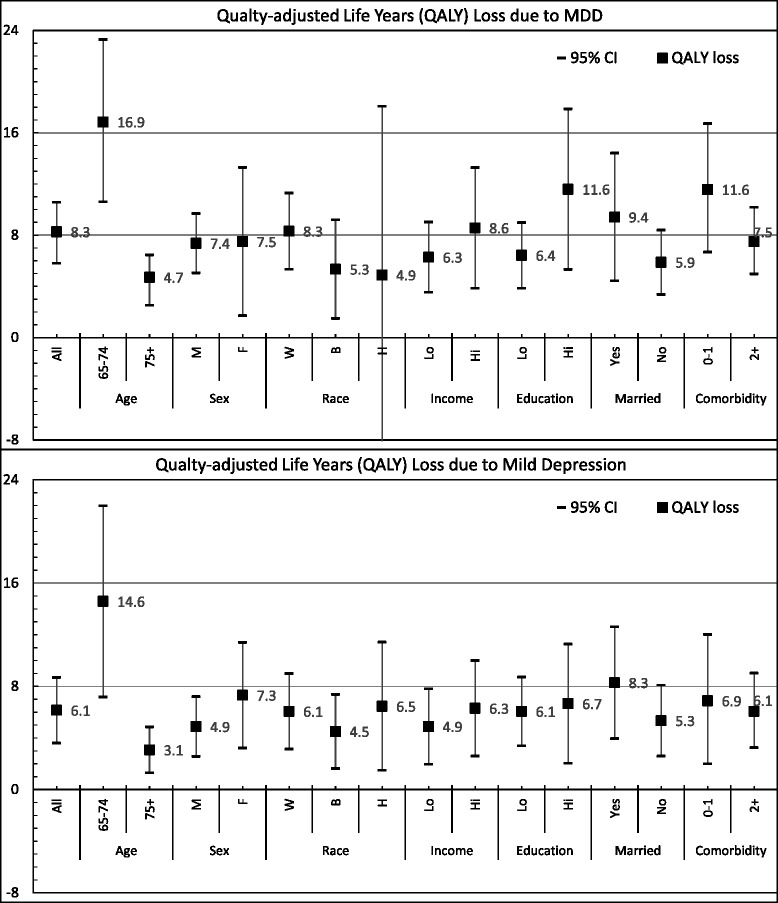
Quality-Adjusted Life Years (QALY) Loss Due to Major Depressive Disorder (MDD) and Mild Depression, Overall and by Subgroups, U.S. Adults Aged 65 Years and Older. Race: W = Non-Hispanic whites, B = Non-Hispanic blacks, H = Hispanics; Income: lo = <138% Federal Poverty Level (FPL), hi = ≥138% FPL; Education: lo = high school or less, hi = greater than high school; Married: Yes = Married or with a partner, No = Widowed, Divorced, Separated, or Never Married

The same patterns were noted in subgroups. Across subgroups defined by age, sex, race/ethnicity, income, education, marital status, and number of comorbidities, persons with MDD had consistently lower QALYs than persons without MDD and those with mild depression had consistently lower QALYs than those with none or minimal depressive symptoms (Fig. 1). The adverse impact of MDD and mild depression on QALY was 3–4 times larger for persons 65 to 74 years old than for persons 75 years old or older. Specifically, QALY losses due to MDD were 16.9 and 4.7 years for persons 65–74 years old and for persons 75+ years, respectively, and losses due to mild depression were 17.0 and 3.6 years, respectively. Of note, the much larger QALY loss for younger participants was mainly because younger participants had a much larger QALY than older participants.

Compared to men, women had a significantly higher prevalence of MDD and mild depression (Table 4). As illustrated in Fig. 1, women and men lost a similar number of QALYs due to MDD (7.4 and 7.5 years). The QALY loss due to mild depression was higher for women than for men (7.3 and 4.9 years), but the difference in QALY loss due to mild depression between men and women was not statistically significant. With regard to race/ethnicity, Non-Hispanic whites and Non-Hispanic blacks had a significant QALY loss due to MDD (8.3 and 5.3 years) and MDS (6.1 and 4.5 years) compared to their counterparts without MDD. For Hispanics, the QALY loss was statistically significant only due to MDS (5.1 years). Although Hispanics with MDD lost 4.9 QALYs, the loss was not statistically significant compared to Hispanics without MDD.

**Table 4:** Percent with Mild Depression and Major Depressive Disorder (MDD), U.S. Adults Aged 65 Years and Older

	No or minimal depression (*N* = 2,863)		Mild depression (*N* = 494)		Major Depressive disorder (*N* = 173)	
	Percent^a^	S.E.	Percent^a^	S.E.	Percent^a^	S.E.
Total	82.1%	0.8%	13.8%	0.6%	4.1%	0.4%
Age						
65–74	82.0%	1.4%	14.2%	1.1%	3.8%	0.5%
75+	82.3%	1.1%	13.5%	1.0%	4.3%	0.5%
Sex						
Men	86.5%	0.9%	10.2%	0.8%	3.3%	0.4%
Women	78.6%	1.2%	16.6%	1.0%	4.7%	0.6%
Race						
Non-Hispanic whites	82.6%	0.9%	13.5%	0.7%	3.8%	0.4%
Non-Hispanic blacks	81.8%	1.6%	14.5%	1.3%	3.7%	0.8%
Hispanics	76.6%	1.9%	15.5%	1.4%	7.9%	1.1%
Other	78.7%	3.2%	15.4%	2.4%	5.9%	1.3%
Income						
<138% FPL^b^	74.6%	1.7%	17.0%	1.4%	8.4%	0.9%
≥138% FPL	84.1%	0.9%	12.8%	0.7%	3.1%	0.4%
Education						
≤High school	79.0%	1.1%	15.6%	1.0%	5.4%	0.6%
>High school	85.9%	1.2%	11.5%	1.0%	2.6%	0.4%
Married or with partner						
Yes	85.6%	0.9%	11.5%	0.9%	2.9%	0.3%
No^c^	76.6%	1.4%	17.4%	1.1%	6.0%	0.7%
Co-morbidities						
0 or 1	86.4%	1.2%	11.7%	1.0%	2.0%	0.3%
2 or more	79.8%	1.0%	14.9%	0.8%	5.3%	0.6%

^a^Weighted percent, accounted for sampling design, noncoverage, and nonresponse

^b^ Federal Poverty Level, where 138% FPL is the Medicaid income eligibility limit

^c^ Divorced, separated, never married, widowed

Compared to their counterparts, significantly higher depressive symptoms were also found among persons who reported a lower income, lower educational achievement, being divorced, separated, never married, or widowed, and having two or more comorbidities (Table 4). The QALY losses due to MDD and due to mild depression were statistically significant for all subgroups according to income category, education achievement, marital status, and number of comorbidities (Fig. 1).

## Discussion

Depression is a prevalent condition that greatly impacts both morbidity and mortality [1, 2, 7, 8]. Previous studies reported a significant burden of disease for MDD [7, 8], but this is the first study, to our knowledge, to estimate QALY according to the severity levels of depressive symptoms. This study not only confirmed the significant burden of disease for MDD among the U.S. elderly, but also showed an incremental decrease in QALYs with an increasing severity of depressive symptoms as well as significant QALY loss due to mild depression. Specifically, individuals with higher (or more impaired) PHQ-9 scores had significantly fewer QALYs. These findings were replicated according to demographic and socioeconomic subgroups.

Our findings indicate that even mild depression is associated with a substantial loss (44% or 6.2 years) in QALY in the elderly. This loss was of a magnitude similar to having diabetes or heart disease [25]. Among persons 65 years and older, depressive symptoms below the threshold for major depression have been shown to cause a higher risk of progression to depression compared to non-depressed elderly, with greater medical burden, worsened functional status, and both poor subjective health status and social support associated with a higher risk of poor outcome [29]. Mild depression also is associated with chronic illness, and has been shown to be a risk factor for cardiovascular mortality [30]. This would be a particular concern in the elderly population where co-morbidities tend to be more common. Even participants characterized as having mild depressive symptoms have reported serious difficulty with work, home, or social activities related to their symptoms [31] and older adults have reported worsened overall quality of life [32].

The treatment for sub-threshold depression has not been firmly established and, for the PHQ-9, the current guideline is to recommend watchful waiting and a repeat PHQ-9 at follow-up [15]. In 2009 the National Institute for Clinical Excellence noted that one or more of the following interventions might be offered for persons with mild depression: individual self-help based on the principles of cognitive behavioral therapy, computerized cognitive behavioral therapy, and/or a structured group physical activity program [33]. At present, a randomized controlled trial is underway to determine if counselling with low-intensity cognitive behavioral interventions are effective for mild depression [34].

Our study has a number of noteworthy limitations. First, the PHQ-9 is not a clinical diagnostic tool for diagnosing depression but has been most widely used as a screening instrument for estimating the prevalence of depression in the general population [15, 17]. This would generate population estimates that were less accurate and reliable compared to a clinical diagnosis or interview. Second, although the results show different amounts of QALY loss due to MDD and mild depression across subgroups (such as between men and women), the sample size was too small to test any differences between subgroups. Third, the NHANES did not include the preference-based HRQOL questions. We used a mapping algorithm to obtain EQ-5D scores for respondents based on their answers to the four Healthy Days questions. Therefore, estimates of QALY loss would also likely be underestimated due to regression toward the mean [35]. However, a previous study that examined the bias of QALE estimates showed that these underestimations were less than 2.5% [35].

This study used a novel method to estimate mean QALYs throughout the remainder of the lifetime for persons according to level of depressive symptoms. Our analyses showed that QALY estimates were reliable even with a small sample size of approximately 100. Because QALY uses the health utility value to weight years of life lived, it provides a means for calculating the economic costs of depression and for analyzing the cost-effectiveness of treatments, interventions, and policies that target depression and its related risk factors [36, 37]. Furthermore, construction of a single index enables the burden of disease attributable to mild depression and MDD to be compared with other chronic conditions and risky behaviors [25].

## Conclusions

In conclusion, among the U.S. elderly, as the severity of depressive symptoms increased, the burden of disease attributable to depression became greater. Our findings of fewer years of QALY for persons with MDD and mild depression were not only statistically significant but also clinically important. These findings have profound implications not only for clinicians but also for public health authorities when setting health priorities and dealing with mental health problems among the elderly population. Given the aging of the U.S. population and the high prevalence of mild depression and MDD, investigators should continue to develop prevention efforts for at-risk elderly as well as effective interventions among persons with a diagnosis of depression.

## Abbreviations

DALYs: Disability-adjusted life years
DSM: Diagnostic and Statistical Manual of Mental Disorders
HRQOL: Health-related quality of life
ICD: the International Classification of Diseases
MDD: Major depressive disorder
NCHS: National Center for Health Statistics
NDI: the National Death Index
NHANES: National Health and Nutrition Examination Survey (NHANES)
PHQ-9: the 9-item Patient Health Questionnaire
QALE: quality-adjusted life expectancy
QALYs: Quality-adjusted life years
